# Immune efficacy of five novel recombinant *Bordetella bronchiseptica* proteins

**DOI:** 10.1186/s12917-015-0488-4

**Published:** 2015-07-30

**Authors:** Yan Liu, Hui Chen, Qiang Wei, Chenwen Xiao, Quanan Ji, Guolian Bao

**Affiliations:** Animal Husbandry and Veterinary Institute, Zhejiang Academy of Agricultural Sciences, Hangzhou, Zhejiang China

**Keywords:** *Bordetella bronchiseptica*, Recombinant proteins, Immune-protective protein, Subunit vaccine

## Abstract

**Background:**

The Gram-negative pathogen *Bordetella bronchiseptica* causes acute and chronic respiratory infection in a variety of animals. Currently, there is no vaccine to prevent these infections. To identify useful candidate antigens for such a vaccine, five *B. bronchiseptica* genes including amino acid ATP-binding cassette transporter substrate-binding protein (ABC), lipoprotein (PL), outer membrane porin protein (PPP), leu/ile/val-binding protein (BPP), and conserved hypothetical protein (CHP) were cloned and the recombinant proteins were expressed. The immune responses of mice to vaccination with individual recombinant proteins were measured.

**Results:**

Each of the tested recombinant proteins induced a high antibody titer. PPP and PL showed protective indices against challenges with *B. bronchiseptica*. The protection ratios were 62.5 and 50 %, respectively, compared with 12.5 % for control vaccinations. The protection ratios of ABC, BPP, and CHP were not significantly different from the controls. IgG-subtype and cytokine analysis demonstrated that PPP and PL can induce two immune responses: a humoral immune response and a cell-mediated immune response. The humoral immunity-mediated, Th2-type response dominated.

**Conclusion:**

The identification of PPP and PL, which offer immune-protective potential, identifies them as candidates for the development of a diagnostic test or a vaccine for *B. bronchiseptica*.

## Background

*Bordetella bronchiseptica* is an important bacterial pathogen that causes a number of respiratory diseases in livestock and poultry. *B. bronchiseptica* can colonize the host for its entire lifetime, resulting in great economic losses worldwide. Examples of diseases caused by *B. bronchiseptica* include atrophic rhinitis in swine, kennel cough in dogs, and snuffles in rabbits [1]. *B. bronchiseptica* infections are often endemic in commercial rabbitries, where they and difficult to control due to the rapid spread and persistence of the infection [2]. Although many available *B. bronchiseptica* vaccines induce high titers of serum antibodies and offer protection against severe diseases [3], *B. bronchiseptica* is often isolated from animals in vaccinated populations, suggesting that the vaccines are not satisfactory in terms of efficacy and safety [4, 5].

The development of a new vaccine is critical to the prevention and control of *B. bronchiseptica* infection. To identify useful *B. bronchiseptica* antigen candidates for use in new diagnostics or vaccines, we previously performed immunoproteomic analyses to analyze the outer membrane proteins of *B. bronchiseptica* and identified a total of 14 common immunoreactive proteins [6]. Here, we selected five of these newly discovered immunogenic proteins as targets for recombinant prokaryotic expression. We tested the recombinant proteins for immunogenicity and protection against *B. bronchiseptica* in mice to find novel immune-protective antigens.

## Methods

### Bacteria and mice

*B. bronchiseptica* strain HB was isolated from a rabbit with infectious rhinitis. The bacteria were cultured on sheep blood agar (Hangzhou Tianhe Microorganism Reagent Co, Ltd) and in tryptone soya broth (TSB, Oxoid, Basingstoke, England, UK) containing 5 % bovine calf serum in a rotary incubator shaker at a speed of 200 rpm at 37 °C for the extraction of DNA. Female ICR mice (18–22 g) were purchased from the Zhejiang Experimental Animal Center (China) and maintained under standard conditions. All animal protocols were approved by the Institutional Animal Care and Use Committee of *Animal Husbandry and Veterinary Institute, Zhejiang Academy of Agricultural Sciences*. Experiments were performed in accordance with the Regulations for the Administration of Affairs Concerning Experimental Animals (China, 1988) and the Standards for the Administration of Experimental Practices (Zhejiang, China, 2009). All surgeries were performed in accordance with the recommendations proposed by the European Commission (1997), and efforts were made to minimize animal suffering.

### Antigen probability, gene amplification, cloning, and sequencing

Five proteins including amino acid ATP-binding cassette transporter substrate-binding protein (ABC), lipoprotein (PL), outer membrane porin protein (PPP), leu/ile/val-binding protein (BPP), and conserved hypothetical protein (CHP) were selected as targets on the basis of previous studies. The reference sequences were analyzed using VaxiJen v.2.0 (http://www.ddg-pharmfac.net/vaxijen/VaxiJen/VaxiJen.html) to determine their antigenic probability. VaxiJen makes antigenic predictions based on the physicochemical properties of proteins without considering their sequence alignments. Accuracy of its predictions is 70–89 % [7]. A threshold value >0.4 was considered imperative for further analysis.

To generate expression constructs for the candidate proteins, five pairs of specific primers were designed (Table 1) to amplify the corresponding genes. Restriction sites (underlined) for *Nco* I and *Xho* I were added at the 5′ end of each forward and reverse primer, respectively. Target genes encoding the mature, full-length proteins without signal peptide sequences were amplified by PCR, digested with *Nco* I and *Xho* I, and ligated to a pET32a + vector. Correct constructs were confirmed using DNA extraction by alkaline lysis, followed by double digestion and sequencing.

**Table 1:** The primer sequences used to amplify the selected proteins

Primer name	Sequence (5′-3′)	Target gene (reference Gene ID)	PCR amplicon size
ABC-F	GCGCCATGGGCAAGAAAATCACCGCTGTA	ABC (ID:2660093)	789 bp
ABC-R	TATCTCGAGCTTGATGATGTTGGCGCCGAA		
BPP-F	GCGCCATGGGCAACAAGGCATTTCGTTTC	BPP (ID: 2663902)	1125 bp
BPP-R	GCGCTCGAGTTACTTGTACGACGTCTTGCT		
CHP-F	TATCCATGGGCTATCCCAACCGCCGGCTGTAC	CHP (ID: 2660968)	573 bp
CHP-R	TATCTCGAGCTACTTGCCGCGCCCGCCTTG		
PL-F	GCGCCATGGGCCGTATGAACAAACGTCAT	PL (ID: 2662792)	1125 bp
PL-R	GCGCTCGAGTCAGACCATCTTGTCCTTCAG		
PPP-F	GCGCCATGGGCAAAAAGACTCTGCTCGCT	PPP (ID: 2660513)	1164 bp
PPP-R	GCGCTCGAGTTAGAAGCGGTGACGGATACC		

The restriction sites are underlined

### Expression and purification of recombinant proteins

The verified constructs were transformed into *Escherichia coli* BL21 cells (DE3, Shanghai, China) for protein expression. Expression of the recombinant proteins was completed according to the manufacturer’s instructions. Briefly, the recombinant proteins were produced as inclusion bodies and purified under denaturing conditions. Purity of the recombinant proteins was determined using 5 % stacking/12 % resolving SDS polyacrylamide gel electrophoresis followed by western blot analysis. The recombinant proteins from non-stained gels were transferred to polyvinylidene fluoride membranes with a semidry transfer apparatus for 45 min at 16 V. Next, the membranes were blocked by incubation with 5 % nonfat milk in phosphate-buffered saline-Tween (PBST) (pH 7.4) overnight at 4 °C. After three washes with PBST, the membranes were incubated with pooled convalescent sera diluted with PBST (1:1000) containing 1 % nonfat milk under gentle agitation at room temperature for 1 h [6]. The membranes were then rinsed in PBST for 15 min and exposed to goat anti-rabbit IgG-horseradish peroxidase (1:5000). Incubation was performed for 1 h at room temperature. The membrane was then washed with PBST three times for 10 min and developed with diaminobenzine for 3 to 5 min to optimize the image.

### Immune responses of mice to recombinant protein vaccination

A total of 48 ICR mice were divided into six groups. Five groups were inoculated with the recombinant proteins (50 μg/dose), respectively, in a 200 μL volume mixed with Freund’s complete adjuvant. A pre-experiment was carried out to define the dose, and we found that immune efficacy of 50 μg/dose rPL was better than 25 μg/dose. A control group was inoculated with 200 μL PBS. Each group was inoculated twice at a 2-week interval. Two weeks after the second inoculation, the mice were challenged intraperitoneally with 1.74 × 10^7^ CFU of *B. bronchiseptica* HB (LD_50_ = 2.42 × 10^6^ CFU). Prior to challenge, serum was collected from each mice via tail bleeding. Antibody titers were measured using indirect enzyme-linked immunosorbent assay (ELISA). Briefly, the purified recombinant proteins were diluted to 1 μg/mL in carbonate–bicarbonate buffer (pH 9.6) and used to coat the wells of a polystyrene plate (100 μL/well). The plates were blocked with 5 % skim milk and incubated with sera from the inoculated mice (1:100 dilutions). The bound antibodies were detected by anti-mouse IgG (1:5000) HRP-conjugated secondary antibody (Abcam, Cambridge, UK) and immune complexes were revealed using the TMB substrate. The antibody responses to the immunizations were determined based on measuring the absorbance at 450 nm in an ELISA reader (Bio-Rad, USA). The protective efficacy was determined by mouse survival up to 10 days post challenge. For the acquired immune-protective proteins, further tests were conducted to determine antibody subtypes IgG1 and IgG2a. To evaluate IgG subclasses (IgG1 and IgG2a), an indirect ELISA was performed similarly to the previous ELISA of IgG. Sera from mice were assessed individually, and all reactions were performed in triplicate.

### Splenocyte proliferation and cytokine analysis of immune-protective proteins

Mice (3 mice/group) were injected with two doses (day 0, 14) of the immune-protective protein rPPP and rPL (50 μg/dose) in a 200 μL volume mixed with Freund’s complete adjuvant, a control group (*n* = 3) was inoculated with 200 μL PBS. All mice of each group were sacrificed under ethyl ether anesthesia 3 weeks after the second inoculation, splenocyte suspensions were prepared and used for lymphocyte proliferation testing (determined by the MTT method). Briefly, 5 × 10^6^ cells/well were cultured in 96-well plates in DMEM supplemented with 10 % FCS. Cells were stimulated with the individual recombinant proteins (10 μg/mL) or medium alone (negative control) at 37 °C with 5 % CO_2_. After 68 h, 20 μL 3-(4,5-dimethylthiazol-2-yl)-2,5- diphenyltetrazolium bromide (MTT, Sigma, St. Louis, US) solution (5 mg/mL) was added to each well and incubated for 4 h. The supernatant fraction of each well was discarded and 100 μl DMSO was added to each well. Absorbance was read at 570 nm using an ELISA reader. The stimulation index (SI) was calculated for each sample. SI is defined as the ratio of the average OD_570_ value samples containing antigen-stimulated cells to the average OD_570_ value of the control cells cultured in medium alone. For the detection of cytokine responses to the acquired immune-protective proteins, splenocytes from the immunized mice were cultured with each recombinant protein as described in the lymphocyte proliferation assay. The concentrations of interleukin-2 (IL-2), IL-4, IL-10, and interferon-gamma (IFN-γ) were measured using a commercial ELISA kit according to the manufacturer’s instructions (R&D, USA). All assays were performed in triplicate.

### Statistical analysis

All data were reported as the mean ± standard error. To determine the significance of the observations, Student’s t-tests were carried out using the SPSS 17.0 software (SPSS Inc., Chicago, US). P values ≤ 0.05 were considered statistically significant.

## Results

### Antigen probability, cloning, expression, and purification of recombinant proteins

**Fig. 1:**
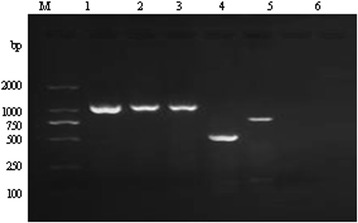
PCR amplification of the target genes from* B. bronchiseptica*. The lanes in the image are as follows: M, DL2000 DNA marker. 1, amplified PPP gene product. 2, amplified BPP gene product. 3, amplified PL gene product. 4, amplified CHP gene product. 5, amplified ABC gene product. 6, negative control

**Fig. 2:**
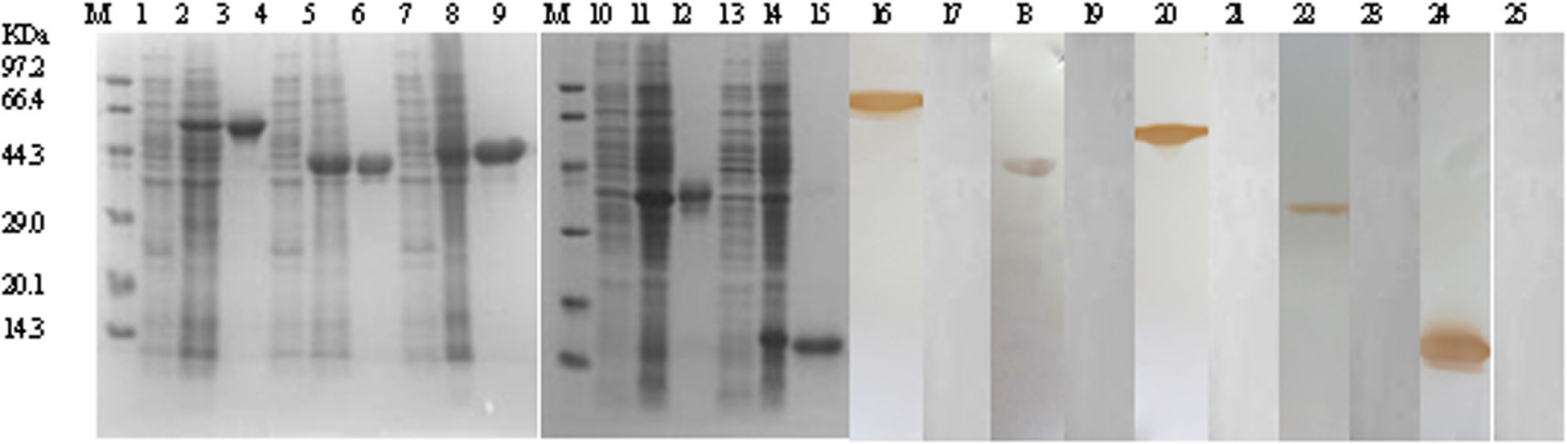
Expression and western blot analysis of recombinant target proteins. Lanes are as follows: Lane M, protein molecular weight marker. Lanes 1–2, 4–5, 7–8, 10–11, 13–14: CBB staining of the recombinant *E. coli* whole-cell lysate before and after induction with IPTG for recombinant ABC, BPP, PPP, PL, and CHP, respectively. Lanes 3, 6, 9, 12, 15: purified recombinant ABC, BPP, PPP, PL, and CHP, respectively. Lanes 16–17, 18–19, 20–21, 22–23, 24–25: western blot analysis of the purified recombinant ABC, BPP, PPP, PL, and CHP with convalescent sera of rabbits before and after infection with *B. bronchiseptica* HB, respectively

The antigenicities of the five proteins were predicted as follows: ABC, 0.6470; BPP, 0.6318; CHP, 0.4716; PL, 0.6523; PPP, 0.6024. Hence, each protein was considered a possible protective antigen and subunit vaccine. The results of the double digestions of the positive plasmids containing the five selected proteins are shown in Fig. 1. Sequence analysis showed that the homology between the amplified genes and the corresponding reference sequences reached more than 97 %, which was consistent with the deduced amino acid sequences. The stripe sizes of the proteins after the plasmids were transformed and expressed in *E. coli* BL21 (DE3) were: rABC, 56.6 KDa; rBPP, 39.7 KDa; rCHP, 21.6 KDa; rPL, 41.5 KDa; rPPP, 41.3 KDa. The recombinant fusion proteins were expressed mainly as insoluble proteins. Thus, the recombinant proteins were solubilized using 8 M urea in lysis buffer. Immunoblot analysis indicated that the recombinant proteins reacted with convalescent sera against *B. bronchiseptica* HB but did not react with negative control serum from uninfected rabbits (Fig. 2).

### Immune responses of mice to recombinant protein vaccination

**Fig. 3:**
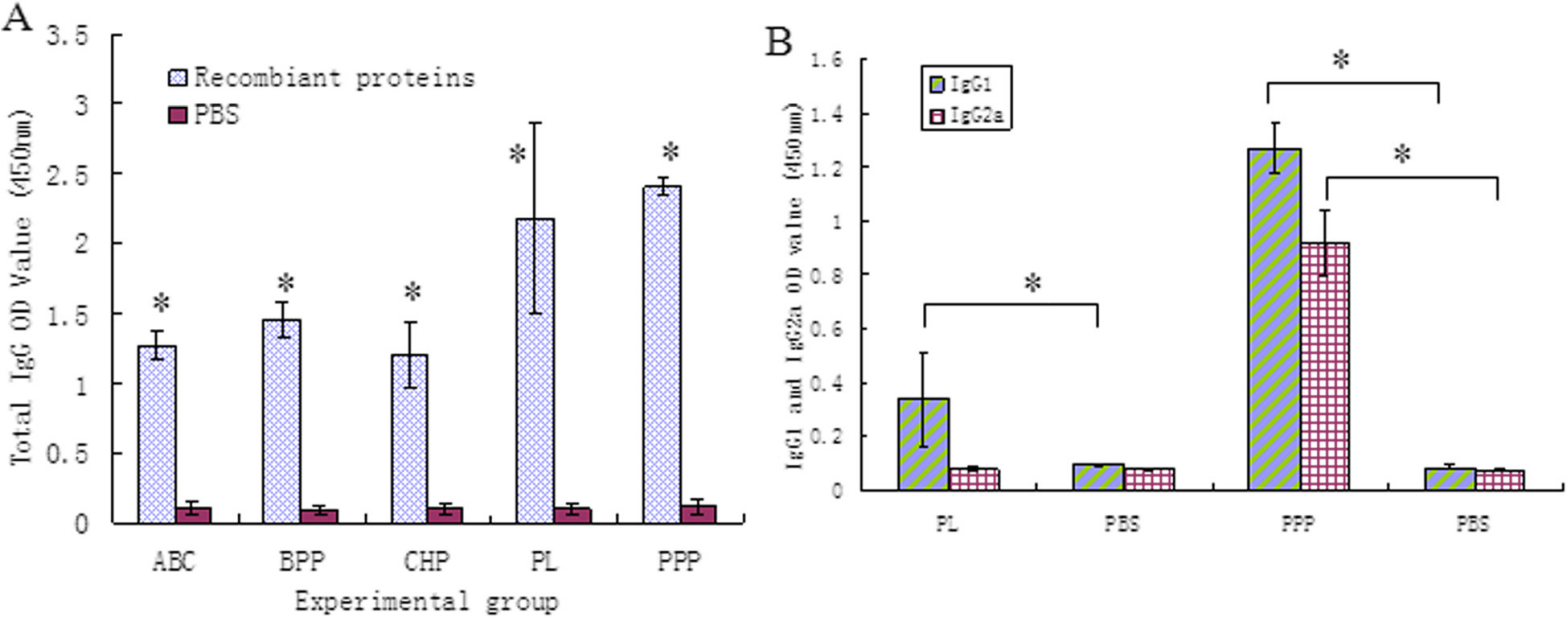
Antibody response elicited after immunization with recombinant proteins or mock-vaccinated (PBS control) before challenge. **a** Histogram indicating the recombinant protein specific serum antibody titers (total IgG) of sera collected from all mice 28 days after first immunization. **b** Histogram indicating the recombinant protein specific serum antibody titers (subtypes IgG1and IgG2a) of sera collected from the mice immunized with rPL, rPPP, and PBS controls at 28 days after first immunization. Data are presented as the mean optical density obtained from ELISA analysis of individual serum samples (*n* = 8 per treatment) at a 1:200 dilution. The curve of antibody response was drawn where the ordinate axis represents the absorbance at 450 nm ± standard deviation. * indicates highly significant differences compared with the mice immunized with PBS (*P* < 0.001)

**Fig. 4:**
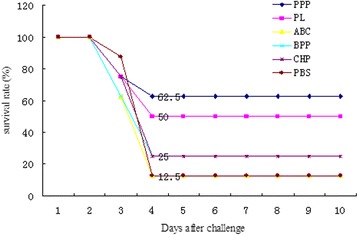
Protective efficacy of recombinant proteins in the mouse model. The percentage survival curve showing survival pattern of immunized and control mice (8 mice/group) following challenge with 1.74 × 10^7^ cfu/mL *B. bronchiseptica* HB, respectively

The antibody titers of mice vaccinated with the recombinant proteins were determined (Fig. 3a). There was a significant increase (*P* < 0.005) in antibodies to the recombinant proteins on day 17 before *B. bronchiseptica* challenge for groups 1, 2, 3, 4, and 5 compared with the control (group 6). Up to 10 days after challenge with *B. bronchiseptica* HB, rPPP and rPL provided better protection [protection ratio of 62.5 % (5/8) and 50 % (4/8), respectively] compared with PBS alone [protection ratio of 12.5 % (1/8); Fig. 4]. No statistically significant differences were observed between the protection ratios of rABC (1/8), rBPP (2/8), or rCHP (1/8), and that of the PBS control. Viable *B. bronchiseptica* was recovered from all the mice that died. For the acquired immune-protective rPPP and rPL, further tests were conducted to determine antibody subtypes. Mice immunized with rPPP produced high titers of specific IgG1 and IgG2a antibodies. The specific IgG1 titer was higher than the specific IgG2a titer (IgG1:IgG2a = 1.38) (Fig. 3b). The mice immunized with rPL mainly produced high titers of IgG1 but low titers of IgG2a (IgG1:IgG2a = 4.2) (Fig. 3b). Examination of IgG subclasses showed that mice responded to recombinant proteins with a Th2-type immune response, which is associated with the stimulation of high IgG1 antibodies and low titres of IgG2, indicating a stronger humoral response.

### Splenocyte proliferation and cytokine analysis of immune-protective proteins

Lymphocyte proliferation assays showed that rPPP and rPL effectively stimulated lymphocyte proliferation (Table 2). Levels of IFN-γ, IL-2, and IL-4 were significantly increased after stimulation by rPPP and rPL. rPL enhanced IL-10 production. rPPP had no significant effect on IL-10 production. The detection of cytokines showed that rPPP and rPL each induced both a humoral immune response and a cell-mediated immune response in the immunized mice.

**Table 2:** Splenocyte proliferation and cytokine analysis of immune-protective proteins

Group (*n* = 3)	Cytokine concentration (pg/ml)^a^				SI^b^
	IFN- γ	IL-2	IL-4	IL-10	
rPPP	625.92 ± 5.91*	18.54 ± 1.41*	202.11 ± 7.37*	320.67 ± 8.96	1.47 ± 0.11
rPL	676.07 ± 5.52*	17.91 ± 0.28*	233.19 ± 13.10*	452.35 ± 12.91*	1.59 ± 0.13
Negative control	546.97 ± 9.39	14.76 ± 0.97	121.81 ± 6.60	288.66 ± 3.60	0.81 ± 0.08

Splenocytes were harvested from the mice 2 weeks after the second immunization with rPPP or rPL. Data represent the mean ± SD from three independent experiments; *n* = 3 mice per group. Single-cell suspensions of splenocytes were stimulated with an optimized concentration of corresponding recombinant protein

*P < 0.05 compared to the control groups

^a^Values for IL-2, IL-4, IL-10, and IFN-γ are titers at 60 h

^b^SI stands for stimulation index

## Discussion

*B. bronchiseptica* is an important pathogen responsible for infectious rhinitis in rabbits, as well as diseases in other animals. The protective effectiveness of the *B. bronchiseptica* vaccine currently used is not satisfactory. Therefore, the identification of antigenic proteins for a new vaccine is urgently needed. Subunit vaccines are attractive and useful tools for the development of protective immunity against pathogenic microorganisms [7, 8]. A number of *B. bronchiseptica* outer membrane proteins have been identified, including filamentous hemagglutinin, pertussis adhesin, adenylyl cyclase hemolysin, tracheal colonization factor, iron-regulating outer membrane protein, outer membrane protein A, lipoprotein, and others. Among these proteins, filamentous hemagglutinin and the outer membrane protein pertactin are factors important for attachment during infection. They also serve as protective immunogens and are among the most important components of acellular vaccines for whooping cough [9]. Previously, our immunoproteomics approach helped us identify 14 common immunogenic *B. bronchiseptica* proteins [6]. Five of those proteins (three putative proteins, ABC, CHP, and PL) were selected and successfully expressed in a recombinant prokaryotic expression system. Western blot analysis indicated that all five recombinant proteins were immunogenic in mice, inducing high antibody titers. Furthermore, PPP and PL induced protective immunity against virulent *B. bronchiseptica* HB in mice.

PPP is an outer membrane porin protein, also known as a channel protein or protein matrix, found in Gram-negative bacteria [10]. Outer membrane porin proteins are highly conserved and can be used for species identification. They display good immunogenicity, making them potential candidates for protective antigens [11, 12]. Previous research supports the hypothesis that porins are good potential subunit vaccine candidates. Mice immunized with purified porin proteins were shown to be resistant to *Pseudomonas aeruginosa* [10]. Research using murine models showed that denatured forms of the recombinant outer membrane porin proteins OmpC, OmpF, and OmpL from *Salmonella enterica* ssp. Typhi were immunogenic [13–15]. In contrast, another study indicated that, despite their strong immunogenicity, these porin proteins are not suitable vaccine candidates in their denatured form; however, a combination of the three recombinant proteins conferred protection [16]. Recombinant OmpF (a channel protein) from *Aeromonas hydrophila* was also reported to have potential as a vaccine candidate, because it could induce a Th1-type immune response. Our results show that rPPP, a novel target antigen derived from an outer membrane porin in *B. bronchiseptica*, can stimulate splenocytes *in vitro* and elicits humoral and cellular immune responses to confer significant protection in mice against challenge by a virulent strain of *B. bronchiseptica*.

PL was identified as a lipoprotein in our previous study. Lipoproteins abound in the outer membranes of Gram-negative bacteria and have long been considered potential target antigens for vaccine development. Hatfaludi *et al*. screened 71 *Pasteurella multocida* proteins and found that only the outer membrane lipoprotein plpE could produce a protective immune response in a denatured state [17]. Another recent analysis suggested that recombinant VacJ lipoprotein from *P. multocida* is a putative vaccine candidate [18]. Our results are the first to demonstrate the immunogenicity and protective efficacy of a putative outer membrane lipoprotein from *B. bronchiseptica*. IgG-subtype and cytokine analysis demonstrated that rPL induced humoral and cell-mediated immune responses in mice and that Th2-type immunity dominates response profile. Th2- type responses have higher levels of IL-10 and produce more IgG1 [19–21]. In the present study, rPL responses have higher levels of IL-10 and produce more IgG1,induced a stronger Th2-type response compared to rPPP. The most direct way to evaluate a vaccine candidate is to measure the survival rate of vaccinated mice challenged with the lethal pathogen. In this study, an effective degree of protection was observed when mice were immunized with rPL compared with controls, suggesting that rPL is an effective vaccine candidate.

Leu/ile/val-binding protein is a component of the leucine, isoleucine, valine, threonine transport system. This system is one of two periplasmic binding-protein-dependent transport systems used for the high-affinity transport of the branched-chain amino acids. Leu/ile/val-binding proteins isolated from *Bordetella pertussis*, *Shigella flexneri*, and *Brucella abortus* have been shown to be highly immunogenic [22–24]. Putative amino acid ABC transporter substrate-binding protein belongs to the ATP-binding cassette (ABC) system, which generally functions to import or export amino acids. ABC systems are versatile, ATP-dependent transport systems for molecules ranging from small inorganic ions to large proteins [25, 26]. ABC systems play important roles in pathogen survival and pathogenicity and have been identified as potential vaccine targets [27]. We cloned and expressed recombinant leu/ile/val-binding protein, amino acid ABC transporter substrate-binding protein, and another conserved hypothetical protein and found that each protein induced high antibody titers but could not elicit significant protective immunity, at least individually in denatured form. A highly immunogenic protein is not necessarily the immune protective protein. For example, the *Leishmania* HSP20 is antigenic during natural infections, but as DNA vaccine, it does not protect BALB/c mice against experimental *L. amazonensis* infection [28]. Furthermore, the results showed here also do not preclude a possible use of the proteins within a multivalent vaccine. In particular, a vaccine combination can induced a better protection [16, 29, 30].

## Conclusion

In conclusion, this study shows that rPPP and rPL can induce protective humoral and cellular immune responses against *B. bronchiseptica*. These data suggest that rPPP and rPL are potential immunomodulators or vaccine candidates if different vaccination approaches such as prime-and-boost vaccination or multivalent subunit vaccines are followed.

## Abbreviations

ABC: Amino acid ATP-binding cassette transporter substrate-binding protein
PL: Lipoprotein
PPP: Outer membrane porin protein
BPP: Leu/ile/val-binding protein
CHP: Conserved hypothetical protein
ELISA: Enzyme-linked immunosorbent assay
SI: Stimulation index
PBS: Phosphate buffered saline
PBST: Phosphate-buffered saline-tween
PCR: Polymerase chain reaction
IPTG: Isopropyl-β-d-thiogalactoside
SDS-PAGE: Sodium dodecyl sulfate-polyacrylamide gel electrophoresis
HRP: Horseradish peroxidase
LD50: Median lethal dose
DMEM: Dulbecco’s modified eagle medium
FCS: Fetal calf serum
MTT: Thiazolyl blue
DMSO: Dimethylsulfoxide
TMB: Tetramethylbenzidine
